# Understanding the effects of beetroot juice intake on CrossFit performance by assessing hormonal, metabolic and mechanical response: a randomized, double-blind, crossover design

**DOI:** 10.1186/s12970-020-00388-z

**Published:** 2020-11-13

**Authors:** Manuel Vicente Garnacho-Castaño, Guillem Palau-Salvà, Noemí Serra-Payá, Mario Ruiz-Hermosel, Marina Berbell, Xavier Viñals, Manuel Gomis Bataller, Teresa Carbonell, Sergio Vilches-Saez, Eulogio Pleguezuelos Cobo, Lorena Molina-Raya

**Affiliations:** 1 GRI-AFIRS. School of Health Sciences, TecnoCampus-Pompeu Fabra University, Ernest Lluch, 32 (Porta Laietana), Mataró, 08302 Barcelona, Spain; 2 Department of Cell Biology, Physiology and Immunology, University of Barcelona, Avda Diagonal 643, 08028 Barcelona, Spain; 3 Physical Medicine and Rehabilitation Department, Hospital de Mataró, Mataró, Barcelona, Spain; 4 Campus Docent Sant Joan de Déu. Fundación Privada, Barcelona, Spain

**Keywords:** Workouts of the day, Testosterone, Cortisol, Testosterone/cortisol ratio, Lactate, Anaerobic performance, Oxygen saturation, Muscular fatigue, Countermovement jump

## Abstract

**Background:**

Acute beetroot juice (BJ) intake has shown to enhance aerobic and anaerobic performance. However, no studies have evaluated the effects of BJ intake on CrossFit (CF) performance by linking hormonal, metabolic, and mechanical responses. The purpose of this study was to determine the causal physiological association between hormonal, metabolic and mechanical responses, and CF workouts performance after acute BJ intake.

**Methods:**

Twelve well-trained male practitioners undertook a CF workout ﻿after drinking 140 mL of BJ (~ 12.8 mmol NO_3_^−^) or placebo. The two experimental conditions (BJ or placebo) were administered using a randomized, double-blind, crossover design. The CF workout consisted of repeating the same exercise routine twice: Wall ball (WB) shots plus full back squat (FBS) with 3-min rest (1st routine) or without rest (2nd routine) between the two exercises. A 3-min rest was established between the two exercise routines.

**Results:**

An interaction effect was observed in the number of repetitions performed (*p* = 0.04). The Bonferroni test determined a higher number of repetitions after BJ than placebo intake when a 3-min rest between WB and FBS (1st routine) was established (*p* = 0.007). An interaction effect was detected in cortisol response (*p* = 0.04). Cortisol showed a higher increase after BJ compared to placebo intake (76% vs. 36%, respectively). No interaction effect was observed in the testosterone and testosterone/cortisol ratio (*p* > 0.05). A significant interaction effect was found in oxygen saturation (*p* = 0.01). A greater oxygen saturation drop was observed in BJ compared to placebo (*p* <  0.05). An interaction effect was verified in muscular fatigue (*p* = 0.03) with a higher muscular fatigue being observed with BJ than placebo (*p* = 0.02).

**Conclusions:**

BJ intake improved anaerobic performance only after the recovery time between exercises. This increase in performance in the first routine probably generated greater hypoxia in the muscle mass involved, possibly conditioning post-exercise performance. This was observed with a fall in oxygen saturation and in muscle fatigue measured at the end of the CF workout. The greatest perceived changes in cortisol levels after BJ intake could be attributed to the nitrate-nitrite-nitric oxide pathway.

## Background

CrossFit® (CF) is considered a strength and conditioning training method characterized by short and high intensity daily sessions called “workouts of the day” (WOD) [1]. CF is based on three modalities such as metabolic conditioning, gymnastics and weightlifting according ﻿to the contents of the WOD [2, 3]. Resistance exercises, gymnastic exercises and aerobic modalities (i.e., rowing, running, or cycling) are combined into a single WOD for improving physical condition [4, 5]. The purpose of WOD is to achieve the best time possible or the largest number of rounds and repetitions within a given time domain [6]. Therefore, performance requirements in CF are primarily linked to improvements in anaerobic power and aerobic capacity [7].

Beetroot juice (BJ) is considered a scientific evidence-based nutritional supplement [8] for improving aerobic and anaerobic performance [9, 10]. This performance enhancement occurs due to the action of the inorganic nitrate (NO_3_^−^) present in BJ. Once ingested, approximately 25% of NO_3_^−^ existent in the mouth is reduced by NO_3_^−^ reductase produced by microorganisms [11] to nitrite (NO_2_^−^) [12]. As it reaches the stomach, NO_2_^−^ is partially reduced to nitric oxide (NO) by the action of stomach acids and is subsequently absorbed in the gut passing into the bloodstream. In conditions of low oxygen levels, the NO_2_^−^﻿ present in plasma could be reduced to NO [12].

NO is a gaseous signaling molecule that plays a determining role in various physiological, hemodynamic and metabolic processes [13]. The most relevant functions include an increase in blood flow into the muscle, reduced oxygen uptake (VO_2_) at a given work rate [14], and blood vessel dilation through mediation by guanylate cyclase [15], among others. Furthermore, NO regulates muscle contraction [16], muscle glucose uptake [17] and mitochondrial biogenesis [18]. All of these functions attributed to NO contribute to optimizing aerobic and anaerobic performance [9, 10].

The assumption that BJ improves aerobic and anaerobic performance is widely debated when referring to well-trained athletes [19]. During exercise in an aerobic domain, Lansley et al. observed that acute dietary NO_3_^−^ supplementation improved time trial performance in ﻿competitive male cyclists [20]. However, our findings determined that acute BJ intake did not improve time trial performance in well-trained triathletes [19]. In anaerobic assessment, peak power was not different between BJ supplementation and placebo (PL) conditions in recreational, competitive and elite sprint athletes in the Wingate test [21]. Other studies have found increases in peak power levels during the Wingate test [22]. The findings of these studies have led to discrepancies about the possible ergogenic effect of BJ in well-trained athletes.

In CF workouts, Kramer et al. demonstrated that chronic dietary NO_3_^−^ supplementation did not improve performance in a specific WOD (Grace workout) and a rowing time trial in trained practitioners [23]. To our knowledge, no more studies have investigated the effects of BJ intake on performance of specific WOD [23], therefore, no accurate conclusions can be drawn about the possible ergogenic effect of BJ intake on CF performance.

On the other hand, it has been documented that NO is an inter- and intracellular messenger for the regulation of some cellular functions, including variations in hormone secretion [24, 25] for anabolic and catabolic purposes. It has been suggested that NO is one of the main stress-mediators involved in the acute response of the hypothalamic-pituitary-adrenal (HPA) axis to exercise. The influence of NO on the pituitary and adrenal cortex has been verified [26], and it has been reported that cortisol secretion could be directly stimulated by NO concentrations after tadalafil administration [27]. Activation of the HPA axis during high-intensity exercise in humans induces cortisol elevation [28] which, as a metabolic and catabolic hormone [29], increases the availability of all fuel substrates by mobilizing glucose [30, 31], amino acids from endogenous stores [32, 33] and free fatty acids [34].

NO also plays a key role in anabolic hormones. Valenti et al. [35] showed that NO triggered a biphasic effect on testosterone secretion which was inhibitory at higher NO levels and stimulatory at lower NO concentrations. As with NO, testosterone may stimulate a vasodilator effect [36, 37]. However, testosterone appears to induce vasodilation at concentrations higher than 10 μmol/L, but at lower physiological concentrations NO seems to be involved in the vasodilatory effect of this hormone [38]. It seems that different NO concentrations could vary the hormonal response of cortisol and testosterone.

Mangine et al. showed that several WOD increased testosterone and cortisol levels for 5 weeks, observing variability in the testosterone/cortisol (T/C) ratio. Hormonal secretion was affected by the type of workout, overload and duration [39]. Recently, it has been reported that testosterone and cortisol levels were not different between advanced and recreational CF-trained participants regarding physical active controls [40]. There are scarce studies evaluating acute hormonal responses during CF workouts and, to the best of our knowledge, no study has evaluated the effects of NO_3_^−^intake on testosterone and cortisol response before (pre) and after (post) CF workouts. Perhaps the increase in NO levels through the exogenous NO_3_^−^ - NO_2_^−^ pathway (BJ intake) modulates testosterone and cortisol concentrations during CF workouts; however, this statement has not yet been scientifically corroborated.

This study aimed to determine the causal physiological association between acute hormonal, metabolic and mechanical responses, and CF workouts performance after acute BJ intake. We speculate that BJ intake improves the specific performance of WOD by increasing hormonal response due to the effect of NO via the NO_3_^−^ / NO_2_^−^ pathway. As WOD are performed to the point of exhaustion, this expected performance improvement could lead to increased metabolic and muscular fatigue.

## Methods

### Participants

This investigation was approved by the Institutional Review Board ﻿according to the principles and policies of the Declaration of Helsinki.

Twelve well-trained male CF practitioners (﻿mean ± SD: age = 29.5 ± 4.3 years, body mass = 79.4 ± 5.5 kg, height = 174.1 ± 6.6 cm) volunteered to participate in this study.

Following an explanation of all experimental procedures, and the associated risks and benefits of participation, each participant provided written informed consent to participate. The following inclusion criteria were established: a) More than 2 years of experience in CF; b) Regional, national and/or international competition level; c) one-repetition maximum (1RM) in full back squat (FBS) greater than 120 kg; ﻿d) no cardiovascular, respiratory, metabolic, neurological or orthopedic disorders that could affect test performance; e) ﻿no consumption of drugs or medication; ﻿f) no smoking; and g) ﻿no consumption of any other supplement at the time of the study.

### Study design

**Fig. 1:**
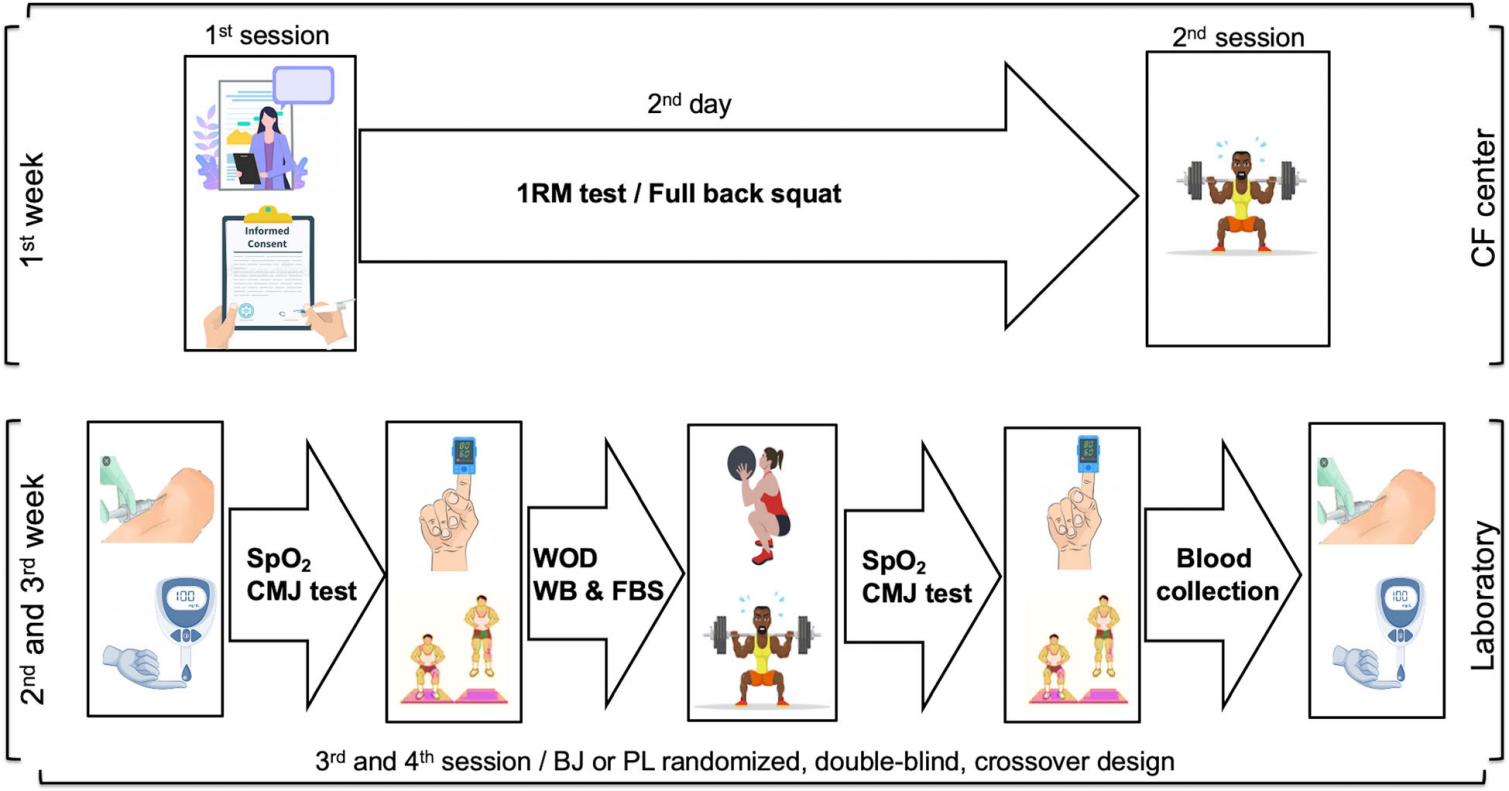
Study design. Abbreviations: 1RM: one-repetition maximum; BJ: beetroot juice; CF: CrossFit; CMJ: countermovement jump; FBS: full back squat; PL: placebo; SpO_2_: arterial oxygen saturation; WB: Wall Ball; WOD: workouts of the day

The procedures applied in the study lasted 3 weeks during which four sessions were performed (Fig. 1). In the first week, participants were required to appear at the CF sports center twice. During the first session, a researcher explained all the experimental procedures and study details to the CF athletes. In the second session, a 1RM test was performed in FBS exercise to determine loading intensity (in kg, 50% of 1RM) that should be used to compare the two experimental conditions.

Over the next 2 weeks, participants completed the WOD test (sessions 3 and 4) at our Exercise Physiology Laboratory to compare the two experimental conditions (BJ and PL). For this purpose, a washout period of 1 week separated each test session. Both sessions were always carried out under similar ambiental conditions (temperature 20 °C– 23 °C, relative humidity 45–55%). The two experimental conditions (BJ or PL) were administered using a randomized, double-blind, crossover design. In each test session, the participants began with the WOD test protocol 3 h after ingesting the BJ or PL. The WOD test consisted of repeating the same exercise routine twice: 1st. Wall ball (WB) shots plus FBS with a 3-min rest between the two exercises. 2nd. WB shots plus FBS without rest between the two exercises. A 3-min rest was established between the two exercise routines.

Before the WOD test, blood collection to determine lactate and hormone concentrations (cortisol and testosterone pretest) were performed under resting conditions upon arrival at the laboratory and 3 h after the BJ or PL intake. Next, arterial oxygen saturation (SpO_2_) and muscular fatigue were assessed (pretest). Once the WOD test was completed, SpO_2_ and muscular fatigue were measured again. After completing SpO_2_ and mechanical fatigue assessment, blood draws were performed to examine lactate and hormone concentrations (post-test), respectively. The participants performed pretest and post-test in both experimental conditions (BJ and PL) at the same time frame (± 30 min) of day.

Participants refrained from any high-intensity physical effort from 72 h and abstained from any type of physical exercise within the previous 24 h before starting the first session to the study end. Participants maintained regular training throughout the study (~ 21 days). They were allowed to perform low intensity workouts, except 24 h before the start of the test. All the participants were completely familiarized with the experimental procedures.

### Diet and beetroot juice intake

The type of diet can condition energy metabolism during exercise [41], therefore, a nutrition professional established nutritional guidelines to ensure that all CF athletes followed a similar diet 48 h before starting the tests which consisted of ~ 60% carbohydrates (5.5 g carbohydrate per kg), 25% lipids and 15% proteins. The diet was registered by the participants 48 h before the first experimental test, and the same diet was replicated 48 h before the second test. Compliance with the established dietary instructions was assessed by checking the participants’ diaries.

Participants were required to avoid foods with a high NO_3_^−^ content at least 72 h before the study outset, and therefore, they were provided with a list of foods (arugula, lettuce, celery, parsley, spinach, turnip, leak, cabbage, endives, beetroot). Intake of caffeine (except breakfast coffee), alcohol, or other supplements was prohibited during the study to avoid any interaction with BJ. Twenty-four hours before the tests, the participants were asked to abstain from brushing their teeth or using a mouthwash and chewing gum or sweets that could contain a bactericidal ingredient such as xylitol or chlorhexidine. Oral antiseptics can alter blood NO_2_^−^ concentrations after NO_3_^−^ intake due to their effects on mouth bacteria [42]. Participants were alerted of possible side effects from BJ intake: a red appearance of urine and feces and gastrointestinal symptoms.

BJ or PL intake occurred 3 h before the start of each test. Scientific evidence indicates that a NO_2_^−^ peak in blood occurs 2–3 h after NO_3_^−^ intake [43]. An expert in nutrition and dietetics prepared the PL drink. The PL drink was prepared by dissolving 2 g of powdered BJ (~ 0.01 mmol, 0.620 mg of NO_3_^−^, Experience-Naturgreen, Murcia, Spain) in a liter of mineral water. Lemon juice was added to simulate the taste of commercial BJ. Participants drank the BJ contained in a randomized assigned bottle containing 140 ml (~ 12.8 mmol, ~ 808 mg NO_3_^−^) of BJ Beet-It-Pro Elite Shot concentrate (Beet IT; James White Drinks Ltd., Ipswich, UK) or PL. Both drinks (BJ and PL) were supplied in an unlabeled 140 ml garnet-red plastic bottle.

### One-repetition maximum full back squat test

﻿ 1RM test protocol was performed as in previous studies [44, 45] according to the guidelines established by Baechle and Earle [46]. The FBS test included a general and specific warm-up for all subjects. The 1RM test was performed with free weight and involved several attempts using increasing weights. The 1RM was defined as the last load (kg) lifted by the participant while completing a knee extension to the required position. The rest period between each attempt was 4 min.

### WOD assessment

After performing a general warm-up consisting of 5 min of low-intensity rowing and 5 min of joint mobility and dynamic stretching exercises, a specific warm-up was carried out consisting of 10 push-ups, 10 walkouts and 10 squat jumps. Next, the WOD test was performed to compare the effects of the two experimental conditions (BJ vs. PL). For this purpose, two characteristic exercises in CF workouts were chosen: WB and FBS.

The WOD test consisted of performing the same exercise routine twice. In the first round, WB shots for 90 s plus FBS for 60 s were completed. A 3-min passive rest (aerobic conditions) was applied between the two exercises because 3–5 min rest allows a greater number of repetitions when performing multiple sets in loads at 50% of 1RM [47]. A 3- to 5-min rest might be safer and more reliable from a psychological and physiological perspective [47]. Next, the same exercise routine was repeated but without recovery time. Then, the second round involved WB shots for 90 s plus FBS for 60 s without rest (anaerobic conditions) between the two exercises. The performance goal was to achieve the highest number of repetitions within a given time domain.

The WOD protocol was designed according to the guidelines established by Glassman [6]. CrossFit workouts can be grouped into three different categories: gymnastics, metabolic conditioning, and weightlifting. The WB and FBS exercises were classified as a weightlifting routine. The principles of “constantly varied, high-intensity, functional movement” were determined as well as a wide variety of mode, exercise, metabolic pathway, rest, intensity, sets and repetitions [6].

### Wall ball technique

A 10 kg medicine ball was used for the WB shots. The WB shots were performed at arm’s length away from the wall starting from the upright position with the knees and hips fully extended with the feet hips-width apart and the toes pointed just slightly outwards, similar to doing a squat. The medicine ball had to be picked up and held at chest-height so that the elbows were tight at the sides. From this position, the butt dropped back and down to lower into a squat while the chest was kept in an upward position with the ball against the sternum. The final position was sitting back onto the heels and trying to achieve as deep a squat as possible. Then, participants immediately reversed the motion and ascended back to the up-right position and simultaneously press, or toss, the ball to a target spot about eight feet up the wall.

### Full back squat technique

FBS exercise was performed with free weight. This was executed starting from the upright position with the knees and hips fully extended. Both feet were positioned flat on the floor in parallel or slightly rotated outwards at a distance of approximately shoulder-width apart. ﻿The barbell ﻿was grasped with a closed pronated grip and placed on the upper back (trapezius muscle) at the level of the acromion. Then, the participants flexed the knees and hips (eccentric action) to descend the barbell in a controlled manner until the top of the thighs reached below the horizontal plane. From this position, the participants immediately reversed motion and ascended back to the up-right position until the knees and hips were fully extended.

### Blood analysis

Participants arrived at the Exercise Physiology Laboratory between 4:00 and 7:00 PM. Blood was collected at the same time of day at rest (pre-test) 3 h after the BJ or PL intake and immediately after intervention (post-test).

Blood was drawn from the antecubital vein into a 10 mL EDTA Vacutainer tube. Next, serum was extracted, centrifuged at 2500 x g for15 minutes, aliquoted and stored at − 80 °C until later analysis. Serum free testosterone levels were determined by the enzyme immunoassay methodology according to the manufacturer’s instructions using the automated Triturus EIA analyzer (Grifols-Quest, Miami, FL, USA). Serum cortisol concentrations were analyzed using chemiluminescent microparticle immunoassay by Abbott Architect immunoassay analyzer (Abbott Laboratories, Abbott Park, IL, USA) [48]. The coefficient of variation for the between and within assay replicates was less than 10%.

Blood lactate concentrations were measured by an experienced investigator using the analyzer Lactate ProTM 2 (Arkray Factory Inc., KDK Corporation, Shiga, Japan). Clean blood samples were obtained from the index finger of the left hand as in previous studies [19, 22]. ﻿The reliability and accuracy of this device has been reported by others [49].

### Arterial oxygen saturation

SpO_2_ was estimated with the Nonin WristOx2™ pulse oximeter (Model 3150, Plymouth, MN, USA). Nonin oximeters have commonly been used for monitoring SpO_2_ [50]. Concretely, the WristOx_2_ 3150 model has an accuracy of ±2% for SpO_2_ measurements [51] and complies with the International Organization for Standardization (ISO) standards ISO 10993-1 and ISO 80601-2-61. SpO_2_ was tested on the index finger of the right hand. Raw data were stored on the internal memory of the WristOx_2_.

### Mechanical fatigue

Lower limb fatigue was determined by a countermovement jump (CMJ) test. The CMJ test was performed using a force platform (Musclelab, Ergotest Technology AS, Langesund, Norway) before and after the WOD test following a method described previously [44, 45]. Participants carried out 3 jumps separated by a 30-s rest, and the mean values of vertical height recorded in the 3 CMJs were used in the statistical analysis.

### Statistical analysis

﻿ The Shapiro-Wilk test was used to check the normal distribution of the data, which are reported as mean and standard deviation (SD), mean and confidence intervals (95% CI) or percentage (%). ﻿To compare the effects of the two experimental conditions (BJ vs. PL), a two-way analysis of variance (ANOVA) with repeated measures was applied (experimental condition x time). ﻿Bonferroni adjustments were used to control for multiple post-hoc comparisons. The magnitude of the response to both experimental conditions was estimated by partial eta-squared (η_p_^2^). The scale for classification of η_p_^2^ was 0.10 = small, 0.25 = medium, 0.40 = large [52]. Statistical power (SP) was calculated.

The formula [(post-test - pretest) / pretest] × 100 was applied to determine the percentage changes (Δ%) between the pretest and the post-test in the measured variables (hormones, lactate, SpO_2_, CMJ). The Student’s t-test for paired data was used to determine significant differences in percentage changes between the two experimental conditions.﻿ Significance was set at *p* <  0.05. All statistical procedures were applied using the software package SPSS version 25.0 for Mac (SPSS Inc., Chicago, IL, USA).

## Results

All participants reported consuming BJ or PL supplement at the correct times. BJ intake was well tolerated by all participants of the study, however, some athletes showed beeturia (red urine) and red stools. The participants maintained exercise and dietary habits before each testing visit according to nutritional and exercise guidelines.

### WOD performance

**Fig. 2:**
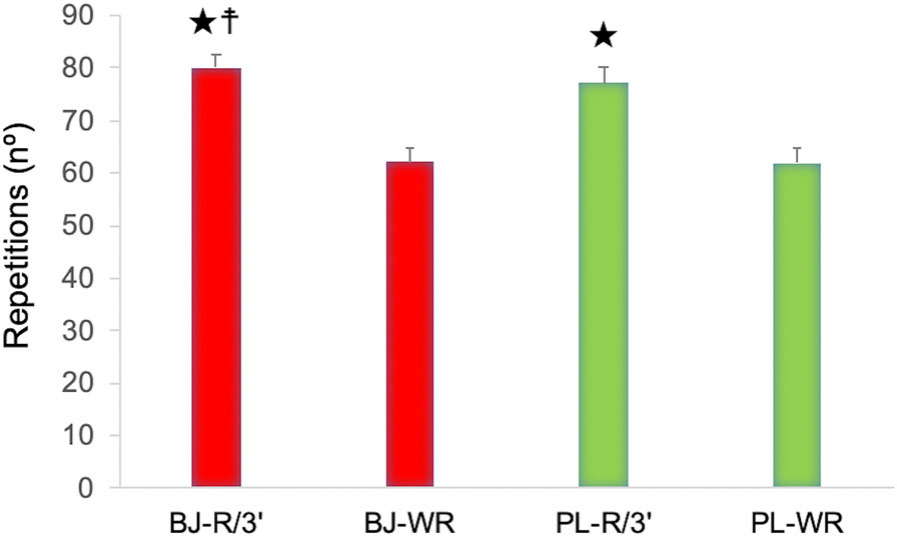
Number of repetitions performed after beetroot juice (BJ) and placebo (PL) intake. Abbreviations: BJ-R/3′: beetroot juice condition with 3-min rest between wall ball shots and full back squat. BJ-WR/3′: beetroot juice condition without rest between wall ball shots and full back squat. PL-R/3′: placebo condition with 3-min rest between wall ball shots and full back squat. PL-WR/3′: placebo condition without rest between wall ball shots and full back squat. ★ A significant increase in the number of repetitions performed in the first routine (with 3-min rest) compared to the second routine (without rest) in both experimental conditions (*p* < 0.001). ☨ A significant increase in the number of repetitions performed in the first routine (with 3-min rest) after BJ intake compared to PL condition (PL-R/3′) (*p* = 0.007). ﻿Data are provided as mean and error bars as 95% confidence intervals

An interaction effect (experimental condition x time) was observed in number of repetitions performed (*p* = 0.04, η_p_^2^ = 0.37, SP = 0.54). A significant effect was detected in the experimental condition and in time (*p* = 0.01, η_p_^2^ = 0.53, SP = 0.80; *p* <  0.001, η_p_^2^ = 0.93, SP = 1.00, respectively). Bonferroni assessment determined a higher number of repetitions in BJ condition than in PL when the rest time between exercises was established in the first routine (*p* = 0.007). However, no significant differences were identified among experimental conditions when recovery time was not applied between exercises in the second routine (*p* > 0.05). A significant decrease in the number of repetitions in the two experimental conditions was observed between the first routine (with recovery time) and the second routine (without rest) (*p* <  0.001) (Fig. 2).

### Serum cortisol and testosterone

**Fig. 3:**
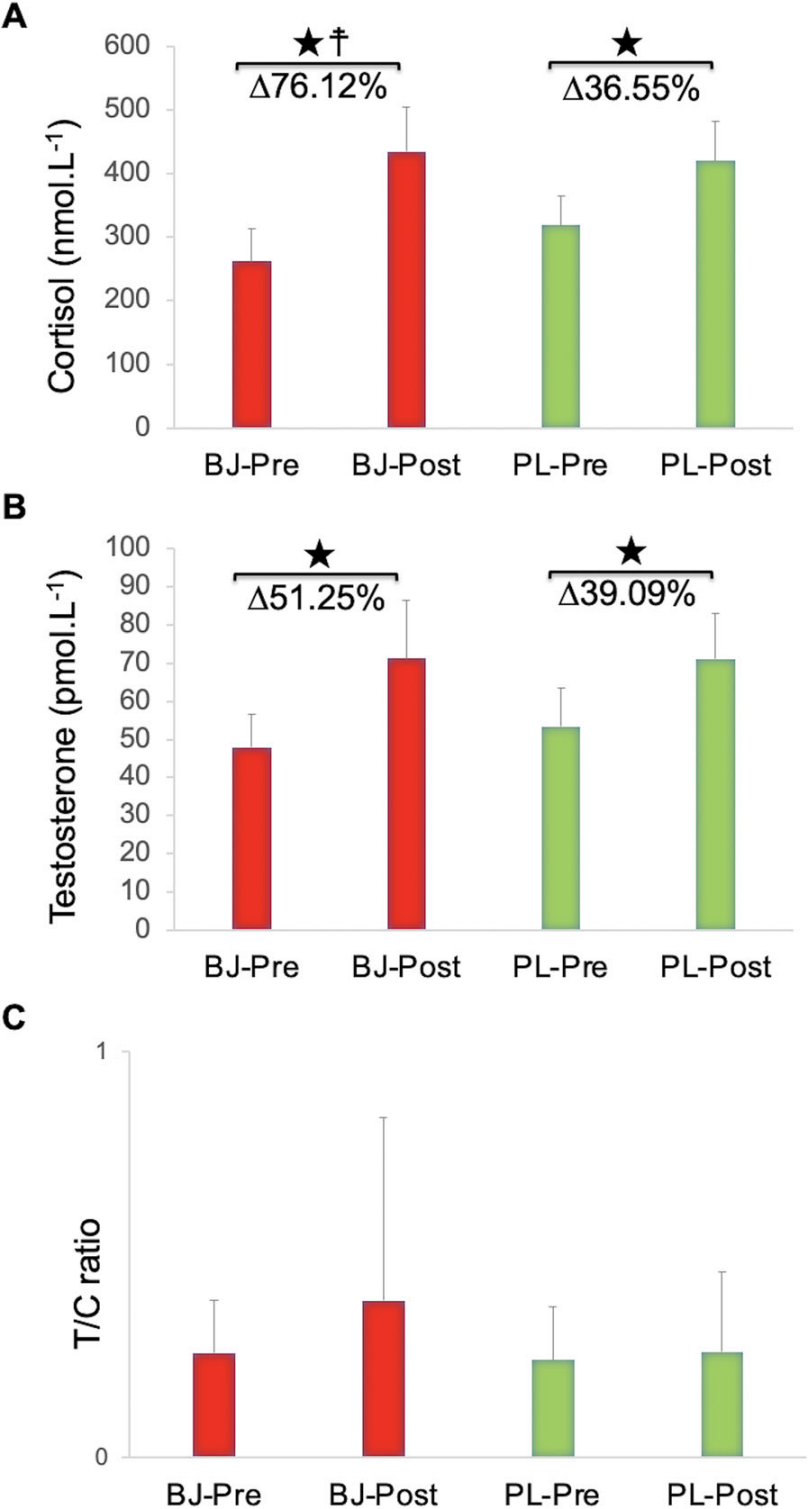
Cortisol (**a**), testosterone (**b**) and testosterone/cortisol ratio (**c**) after beetroot juice and placebo intake. Abbreviations: BJ: beetroot juice; PL: placebo; T/C ratio: testosterone/cortisol ratio. ★ A significant raise in testosterone and cortisol concentrations after WOD test (post-test) compared to pretest in both experimental conditions (*p* < 0.01). ☨ Significantly higher increase (Δ%) in cortisol concentrations after BJ intake compared to PL condition (*p* = 0.004). ﻿Data are provided as mean and error bars as 95% confidence intervals

For serum cortisol, an interaction effect (experimental condition x time) and time effect were verified (*p* = 0.04, η_p_^2^ = 0.32, SP = 0.55; *p* < 0.001, η_p_^2^ = 0.77, SP = 1.00, respectively). No experimental condition effect was found (*p* > 0.05). The Bonferroni test confirmed significantly elevated levels of cortisol in the two experimental groups at the end of the exercise (BJ: *p* < 0.001; PL: *p* = 0.005). Cortisol levels increased to a greater extent (*p* = 0.004) after BJ intake (76.12%) than in the PL condition (36.55%) (Fig. 3a).

For serum testosterone, no interaction effect (experimental condition x time) and experimental condition effect were observed (*p* > 0.05). A significant time effect was found (*p* < 0.001, η_p_^2^ = 0.79, SP = 1.00) (Fig. 3b). No interaction effect (experimental condition x time), time effect or experimental condition effect were observed in the T/C ratio (*p* > 0.05) (Fig. 3c).

### Lactate, oxygen saturation and mechanical fatigue

The statistical analyses corresponding to lactate, SpO_2_ and mechanical fatigue are shown in Table 1.

**Table 1:** Metabolic fatigue (lactate), arterial oxygen saturation (SpO_2_) and mechanical fatigue (CMJ test) produced in both experimental conditions

	BJ-Pre (95% CI)	BJ-Post (95% CI)	PL-Pre (95% CI)	PL-Post (95% CI)	*P*^1^ (ES-SP)	*P*^2^ (ES-SP)	*P*^3^ (ES-SP)
Lactate (mmol. L^−1^)	1.10	18.61^★^	1.06	17.33^★^	0.320	**< 0.001**	0.307
	(0.99–1.20)	(15.89–21.32)	(0.94–1.19)	(16.12–18.54)	(0.10–0.16)	(0.98–1.00)	(0.10–0.16)
SpO_2_ (%)	97.46	94.27^☨^	97.01	94.46^☨^	**0.011**	**< 0.001**	0.539
	(96.59–98.32)	(93.60–94.95)	(96.09–97.90)	(93.83–95.08)	(0.50–0.81)	(0.84–1.00)	(0.04–0.09)
CMJ (cm)	42.42	31.09^☨^	42.21	35.79^☨^	**0.033**	**0.015**	0.281
	(31.53–53.30)	(23.42–38.75)	(35.32–49.10)	(31.35–40.22)	(0.63–0.65)	(0.73–0.82)	(0.23–0.17)

*Abbreviations*: *BJ* beetroot juice, *CMJ* countermovement jump, *ES* effect size, *PL* placebo, *SP* statistical power. *P*^1^-values for experimental condition x time interaction effect. *P*^2^-values for time effect. *P*^3^-values for experimental condition effect. ^★^A significant increase in blood lactate concentrations after WOD test (post-test) compared to pretest in both experimental conditions (*p* < 0.001). ^☨^A significant decrease in SpO_2_ and vertical jump height after WOD test (post-test) compared to pretest in both experimental conditions (*p* < 0.05). Data are provided as mean and 95% confidence intervals (95% CI)

No interaction effect (experimental condition x time) and experimental condition effect were observed in blood lactate concentrations (*p* > 0.05). As expected, a time effect was confirmed in both experimental conditions (*p* < 0.001, η_p_^2^ = 0.98, SP = 1.00).

**Fig. 4:**
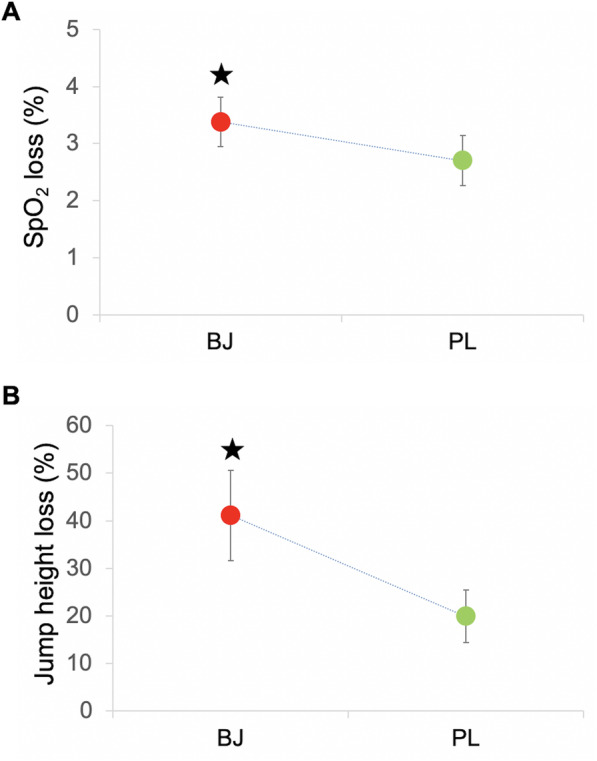
Arterial oxygen saturation (**a**) and jump height (**b**) losses (%). Abbreviations: BJ: beetroot juice; PL: placebo; arterial oxygen saturation: SpO_2_. ★ Significantly higher increase (Δ%) in SpO_2_ loss (*p* = 0.01) and vertical jump height loss (*p* = 0.02) after BJ intake compared to PL condition. ﻿Data are provided as mean and error bars as 95% confidence intervals

For SpO_2_, a significant interaction effect (experimental condition x time) was found (*p* = 0.01, η_p_^2^ = 0.49, SP = 0.81). A significant time effect was identified (*p* < 0.001, η_p_^2^ = 0.84, SP = 1.00). However, no experimental condition effect was produced (*p* > 0.05). The Bonferroni test confirmed a significant fall in SpO_2_ in both experimental conditions at the end of the WOD (*p* < 0.05). A greater SpO_2_ decrease (Δ%) was observed with BJ (3.38%) than PL (2.69%) (*p* = 0.01) (Fig. 4a).

For the CMJ test, a significant interaction effect (experimental condition x time) was determined (*p* = 0.03, η_p_^2^ = 0.63, SP = 0.65), and a significant time effect was identified (*p* = 0.02, η_p_^2^ = 0.73, SP = 0.82), while no experimental condition effect was observed (*p* > 0.05). The Bonferroni test confirmed significant losses in jumping ability in both experimental conditions (*p* < 0.05). A greater decline (Δ%) in vertical jump height was observed with BJ (41.16%) than PL (19.88%) (*p* = 0.02) (Fig. 4b).

## Discussion

The main finding of this study was that BJ intake improved WOD performance only after the rest time between exercises. Interestingly, cortisol levels and muscular fatigue increased, and SpO_2_ fell to a greater extent following BJ intake than under PL conditions. Contrary to our study hypothesis, this improvement in exercise performance was not due to a physiological causal relationship between NO_3_^−^ intake and testosterone concentrations, blood lactate response and the T/C ratio.

There are limited studies available regarding the effect of BJ intake on WOD performance. Kramer et al. analyzed the effects of chronic NO_3_^−^ supplementation on performance during a specific WOD [23]. The WOD was different from that proposed in this study, however, they did not find improvements in WOD performance. Both WOD were characterized by a strong involvement of anaerobic metabolism and the need to maintain the effort required through aerobic metabolism. Surprisingly, we only discovered a positive ergogenic effect of BJ when a 3-min rest was established between the WB shots and the FBS exercise. When WB and FBS exercises were executed without rest, anaerobic conditions of the routine were increased, and no improvements were noted in the number of repetitions.

During rest periods, the capacity to repeat exercise sets at high work rates depends on the rate of phosphocreatine (PCr) resynthesis by oxidative phosphorylation. PCr resynthesis is determined through local muscle blood flow and O_2_ delivery to skeletal muscle [53, 54]. Acidosis resulting from the accumulation of H^+^ inhibits oxidative phosphorylation and may limit adenosine triphosphate (ATP) supply in exercising muscle [55]. An acidified environment (low muscle pH) could increase the availability of NO from NO_2_^−^ during heavy working skeletal muscle [56]. The blood lactate concentrations obtained in this study demonstrated that WOD was performed under high acidification conditions. The muscle mass involved in exercise could provide an environment conducive to the production of NO from NO_2_^−^ [57] especially when muscle oxygenation is poor under lactic anaerobic conditions.

Bearing in mind that production of NO from NO_2_^−^ is exercise intensity-dependent in a predominantly anaerobic environment, BJ intake could delay fatigue by facilitating the regulation of blood flow [12] and local O_2_ supply to active skeletal muscle [57] by increasing circulating NO_2_^−^ levels during rest time (first routine). As an ergogenic aid, BJ intake before training or competition of high intensity intermittent efforts (high lactate levels) could be an appropriate strategy to delay muscle fatigue and improve exercise performance during rest time between sets. Knowledge of the recovery time between exercises or sets would be of interest to assess the time required by NO from NO_2_^−^ to optimize vasodilation and local oxygenation supply to active muscle mass. It seems that at least 3 min of rest is an adequate interval to increase the number of repetitions in the first routine. Further research is warranted to elucidate the potential efficacy of BJ ingestion during recovery time in primarily anaerobic efforts.

As expected, cortisol and testosterone levels were markedly increased in both experimental conditions. CF workouts seem to influence testosterone and cortisol hormonal response [39, 58]. However, there is little scientific evidence to suggest that consistent alterations in cortisol and testosterone concentrations occur after BJ intake.

Cortisol levels augmented in both experimental groups in response to high-intensity exercise. When the percentage change (Δ%) was compared in the two experimental conditions, the BJ condition cortisol levels significantly increased compared to PL (76.12% vs. 36.54%, respectively). It has been stated that cortisol secretion is induced during high-intensity exercise in humans [28] and is also stimulated by NO concentrations [27]. We conjecture that a series of exercise- and BJ-dependent multisystemic physiological events were triggered. The lactic anaerobic workout (blood lactate = ~ 18 mmol. L^− 1^) appreciably altered cortisol response in both experimental conditions during high-intensity exercise [28]. BJ intake raised plasma NO_2_^−^ levels via NO_3_^−^ ingestion to a greater extent than in PL conditions as in previous studies [59, 60]. Hypoxia generated in the muscle mass elicited the NO response via the NO_2_^−^﻿ present in plasma [12]. Increased NO response with BJ intake induced a higher cortisol response [27] (Δ%) than in PL conditions. Cortisol elevation enlarged the availability of all fuel substrates by mobilizing glucose [30, 31]. Consequently, BJ improved performance in the first routine and, notwithstanding increasing anaerobic exercise conditions, performance was maintained during the second routine. ﻿Unfortunately, our arguments cannot be objectively corroborated with data since plasma NO_2_^−^ levels were not measured, ﻿therefore, these observations remain purely intuitive and speculative. However, it is assumed that BJ intake considerably increases plasma NO_2_^−^ levels [60].

Despite the fact that NO influences the hormonal response of testosterone [38], the ingested dose of NO_3_^−^ did not sufficiently stimulate NO concentrations to modify testosterone levels and the T/C ratio. Testosterone response may have been conditioned by the cortisol levels observed after the CF workouts. High cortisol levels in response to anaerobic exercise possibly caused a potential for inhibition of testosterone production via steroid inhibition, and thus, cortisol induced a direct suppressive effect on testosterone steroidogenesis [61].

The T/C ratio has been identified as an indicator of anabolic/catabolic status during exercise [62]. An absolute ratio less than or equal to 0.35 × 10^− 3^ or a decline in the free testosterone to cortisol ratio higher than 30% is suggested to be indicative of a negative catabolic state [63]. Variability in the T/C ratio has been described in response to high-intensity workouts [39]. The data reported in this study did not indicate a negative catabolic state or changes between the two experimental groups. BJ intake did not appear to influence anabolic/catabolic status in response to high intensity workouts.

Another physiological mechanism associated with the low availability of muscle oxygen during lactic anaerobic efforts could be the decrease in SpO_2_. To our knowledge, there are no studies examining the effects of BJ intake on SpO_2_ during CF workouts, thus, the arguments are limited to discussing findings related to aerobic and anaerobic efforts. Several studies reported that BJ did not alter SpO_2_ during predominantly aerobic efforts in trained male cyclists [64] and well-trained runners [65] under normoxia and hypoxia conditions. Engan et al. described an increase in SpO_2_ during a sub-maximal ‘dry’ apnea after acute BJ supplementation (~ 5 mmol NO_3_^−^) [66]. Other authors determined that BJ supplementation did not prompt a significant variation in SpO_2_ during submaximal static and dynamic apneas [67]. The results reported support an interesting and controversial debate about the effects of NO_3_^−^ intake on SpO_2_. Arnold et al. indicated that runners who improved time-trial performance responded to hypoxia with greater arterial desaturation [65]. In our study, CF athletes who improved WOD performance after BJ intake also showed a greater reduction in SpO_2_ (3.4%) than after PL intake (2.7%). A decline in SpO_2_ is indicative of an intensification in oxygen uptake by the muscle mass involved. The greater number of repetitions carried out in the total WOD after BJ intake likely caused an increase in oxygen consumption in the active muscles and promoted a fall in SpO_2_, demanding the participation of lactic anaerobic metabolism.

A predominantly anaerobic metabolism was evidenced in both experimental conditions by the lactate concentrations detected. Rogatzki et al. demonstrated using a parallel squat exercise in which lactate appears to accumulate in response to a rising number of repetitions with reducing recovery time between sets [68]. In their study, a workout of muscular endurance was compared with a hypertrophy workout. Higher blood lactate levels were observed during muscular endurance training (Δ 24.5%). In our study, a higher number of repetitions was carried out during the first exercise routine after BJ conditions. In the second routine, recovery time was suppressed, and a similar number of repetitions was completed (Δ 0.5%, no significant) following BJ ingestion. The total count of repetitions including the first and second routine was significantly higher after BJ intake than in PL condition (data not shown). However, blood lactate levels were 3.1% higher (no significant) after BJ than PL intake. Previous findings by our research group reported that BJ increased power output (during the first 15 s) and lactate concentrations during the Wingate test (82.6%) [22]. On intermittent exercise, other groups have found that lactate (pre-exercise vs. post-exercise) was higher after BJ intake in the 24 × 6-s and 7 × 30-s protocols, but that was not the case in the 6 × 60-s protocol [69].

The debate over the role that BJ plays in lactate response to exercise is controversial. In our study, lactate concentrations almost doubled or tripled those of the studies mentioned. The differences observed among studies might be attributed to the type of exercise and the evaluation protocol used in each study. BJ was expected to increase lactate concentrations to a higher magnitude than under PL conditions, however this physiological event did not occur.

The fall in SpO_2_ and the blood lactate concentrations determined a significant metabolic fatigue in both experimental conditions. Rises in lactate values provides information about a possible state of muscle fatigue [68]. Previous findings in CF practitioners have determined that metabolic fatigue is associated with post-exercise muscular fatigue [70]. Effectively, a higher muscular fatigue was verified after BJ intake (41.16%) than in PL (19.88%) conditions. Mechanical fatigue evaluated by a CMJ test has been considered as a good indicator of muscle fatigue in resistance exercises and in CF workouts in aerobic and anaerobic conditions [70, 71]. We suggest that the physiological benefits (vasodilation, increased blood flow, etc.) of BJ during the established 3-min rest after WB produced an increase in the number of repetitions in the FBS exercise (1st routine). This improvement in performance probably increased the anaerobic conditions of the muscle mass involved before starting the second routine and, therefore, a greater fall in S_P_O_2_ to supply the oxygen needs of the muscle mass was produced at the end of exercise. The relationship of these physiological mechanisms resulted in increased muscle fatigue after completing the WOD.

This study presents some limitations that should be considered. Plasma NO_2_^−^ concentrations were not quantified, thus, our arguments were based on other investigations that found significant increases in plasma NO_2_^−^ levels [59, 60]. More research is needed to confirm an increase in the hormonal response of cortisol via the NO_3_^−^-NO_2_^−^-NO pathway in high intensity exercise.

The sample size should be augmented, because ﻿minimal changes are usually observed in well-trained athletes [19]. Notwithstanding the significant increase in WOD performance (η_p_^2^ = 0.37, SP = 0.54) and cortisol levels (η_p_^2^ = 0.32 and the SP = 0.55), the magnitude of the response observed, and the SP would be increased with a larger sample. An increased cortisol response was observed in nine athletes after BJ intake.

## Conclusions

The findings of this study determined that:

BJ intake improved WOD performance only after a rest time between WB and FBS exercises. Performance was maintained when anaerobic conditions of exercise were augmented.

This performance increase in the first routine generated greater hypoxia in the muscle mass involved, possibly conditioning post-exercise performance. This was observed in the fall in SpO_2_ and in the increase in muscle fatigue measured at the end of the WOD.

The greatest changes observed in cortisol levels after BJ intake might be attributed to the NO_3_^−^ - NO_2_^−^- NO pathway. More research is needed to confirm such assumption.

## Data Availability

Data are presented in the manuscript, further information available upon request.

## Abbreviations

1RM: One-repetition maximum
ANOVA: Analysis of variance
ATP: Adenosine triphosphate
BJ: Beetroot juice
CF: CrossFit
CI: Confidence intervals
CMJ: Countermovement jump
FBS: Full back squat
HPA: Hypothalamic-pituitary-adrenal
NO: Nitric oxid
NO_2_^−^: Nitrite
NO_3_^−^: Nitrate
η_p_^2^: partial eta-squared
PCr: Phosphocreatine
PL: placebo
SP: statistical power
SpO_2_: Arterial oxygen saturation
T/C: Testosterone/cortisol
VO_2_: Oxygen uptake
WB: Wall Ball
WOD: Workouts of the day
